# Economic analysis of triclosan-coated versus uncoated sutures at preventing surgical site infection in patients undergoing abdominal surgery

**DOI:** 10.1136/bmjsit-2025-000383

**Published:** 2025-06-12

**Authors:** Mwayi Kachapila, Dmitri Nepogodiev, Bryar Kadir, Maria Picciochi, Sivesh K Kamarajah, Aneel Bhangu, Raymond Oppong

**Affiliations:** 1 Applied Health Sciences, University of Birmingham–Edgbaston Campus, Birmingham, UK; 2 University of Birmingham, Birmingham, UK

**Keywords:** Health Care Costs, Colon and Rectal Devices, Health Technology, Health Policy, Laparoscopy

## Abstract

**Objectives:**

A recent meta-analysis of high-quality randomized trials casts doubt on the effectiveness of triclosan-coated sutures in reducing surgical site infection (SSI). This economic analysis is aimed at assessing whether triclosan-coated sutures, compared with uncoated sutures, can reduce costs from a healthcare perspective.

**Design:**

This was a model-based economic analysis mainly informed by baseline SSI rates, effect size CIs from a recent meta-analysis of high-quality trials (OR 0.90, 95% CI 0.74 to 1.09, p=0.29), and country-specific cost data.

**Setting:**

This was a worldwide analysis that estimated average cost savings aggregated for high, middle, and low Human Development Index (HDI) countries and country-specific cost savings for the 193 countries on the HDI list.

**Participants:**

Participants were patients undergoing abdominal surgery. The analysis was informed by baseline SSI rates from an international cohort study (12 539 patients).

**Main outcome measures:**

Results are reported in 2022 US dollars as average cost differences associated with SSI between coated and uncoated sutures. Deterministic sensitivity analyses examined variations in suture cost, hospital stay costs, and effect size, with best and worst-case scenario analyses.

**Results:**

SSI-related cost differences per patient ranged from −$466 to $171 in high-HDI, −$23 to $18 in middle-HDI, and −$34 to $22 in low-HDI countries when triclosan-coated sutures were used. The largest potential savings and expenditure occurred in contaminated-dirty wounds. Similar results were observed at the national level in 184 of 193 countries. Best-case to worst-case analyses showed a range of −$533 to $192 in high-HDI, −$57 to $49 in middle-HDI and −$69 to $52 in low-HDI countries.

**Conclusions:**

This analysis highlights significant uncertainty regarding cost savings with routine use of triclosan-coated sutures, emphasizing the need for high-quality data and CI-based economic analysis in policy making.

**WHAT IS ALREADY KNOWN ON THIS TOPIC**

- Triclosan-coated suture has been recommended as a surgical site infection (SSI) prevention method among abdominal surgery patients. However, recent evidence cast doubts on the effectiveness of intervention.

**WHAT THIS STUDY ADDS**

- This model-based economic analysis confirms that there is high uncertainty on the use of triclosan-coated suture, compared with uncoated suture, in reducing SSIs among abdominal surgery patients globally. The intervention can either reduce or increase healthcare costs.

**HOW THIS STUDY MIGHT AFFECT RESEARCH, PRACTICE OR POLICY**

- To establish more conclusive evidence on the effectiveness of triclosan-coated sutures, future trials should employ rigorous methodologies. Furthermore, this study underscores the importance of using the CI approach to better represent the uncertainty inherent in an intervention rather than solely relying on a point estimate.

## Introduction

 Surgical site infection (SSI) is the most common complication after surgery worldwide and affects both patients and healthcare systems.^1^ It affects 9.4% of surgical patients in high Human Development Index (HDI) countries, 14.0% in middle-HDI countries, and 23.2% in low-HDI countries.^2^ Patients experience pain, anxiety, and emotional and social adverse effects, including a decline in mental health and quality of life, which can persist for several months and incur higher direct and indirect healthcare costs compared with patients without SSIs.^3^,^8^ Additionally, patients with SSI have higher societal costs associated with lost income due to late return to work and mortality, which may represent 90% of overall SSI costs.^5^

In the past decade, the WHO recommended 29 strategies for SSI prevention, including chlorhexidine gluconate for skin preparation and triclosan-coated suture for abdominal wall closure.^9 10^ However, evidence that informed the recommendations was not of high quality.^1^ Meta-analyses of randomized trials for triclosan-coated sutures have proved overwhelmingly positive effect but have mostly been based on mixed quality data with a strong dilution effect from low-quality trials. More recent meta-analysis that focused on only five methodologically high-quality randomized trials showed that there was no significant difference in SSI rates between triclosan-coated sutures and uncoated sutures.^11^ The data were from high, middle, and low-HDI countries and numerically outweighed data from low-quality trials, providing a more definitive conclusion.

Previous economic analyses that evaluated the impact of triclosan-coated sutures, compared with uncoated sutures, at SSI prevention included data from Italy, USA, Egypt, and UK among several surgical procedures including abdominal surgery.^12^,^15^ However, economic analysis results, including analyses on SSIs, from one country may not be useful to decision-makers in another country because of differences in SSI rates, wound complications, healthcare systems, and economic conditions across countries.^16 17^ The aim of this economic analysis was to assess the costs associated with SSIs when triclosan-coated suture was used, compared with uncoated suture, among patients undergoing elective and emergency abdominal surgery in all countries in the world. We aimed to use a CI approach, in keeping with statistical decision-making around clinical effects, where interpretation is based around uncertainty from a CI rather than a single point estimate, which is highly likely to be misleading.

## Methods

### Overall study design

This model-based economic analysis was conducted to determine whether triclosan-coated suture use is associated with a net reduction in healthcare costs from a healthcare perspective in high, middle, and low HDIs using the 2022 classifications.^18 19^ The multistep analysis evaluated the average total healthcare costs associated with SSIs in triclosan-coated versus uncoated suture groups in patients undergoing abdominal surgery based on the 95% CI of the effect size of coated suture.^11^ This model focused on the suture-related cost, defined as the fascial suture cost plus the average cost per patient of treating SSI, calculated by multiplying the SSI rate and cost of managing one SSI.

### Patient and public involvement

Patient and public involvement was not appropriate for this study because it was a model-based economic analysis that used secondary data from published studies. As this study used existing data, direct engagement with patients or the public was not necessary. While the study aims at improving the patient outcomes through reduced SSIs and reduced costs, the modeling approach used in the study presented challenges for direct patient and public involvement in that specific aspect of the research process.

### Data sources

**Figure 1:**
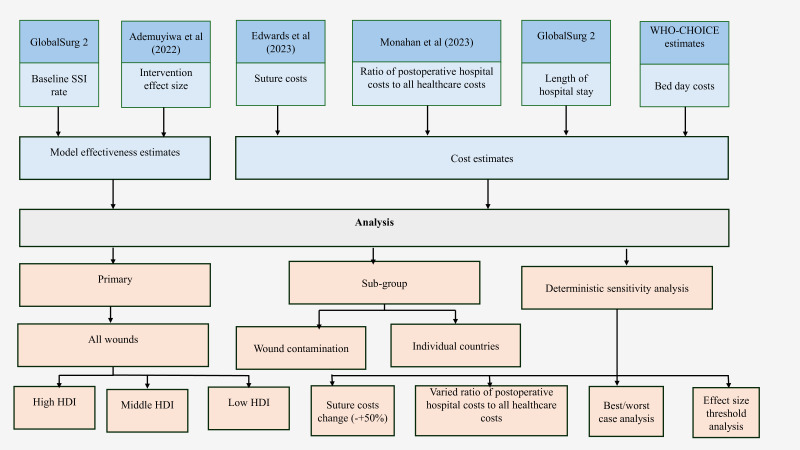
Flow chart showing all data sources used in the model. Threshold analysis is a sensitivity analysis that is conducted to identify the value of a given parameter that alters the base case results in an analysis. Best/worst-case scenario is a sensitivity analysis that examines the combination of the most favorable/unfavorable values of parameters on the results of the analysis. HDI, Human Development Index; SSI, surgical site infection.

To perform the analysis, several data sources (figure 1) were required as below:

1. *Baseline effect size*: The baseline SSI rates for each country were identified from a dataset of a recent international, prospective, multicenter, cohort study. The study measured the incidence of SSI among 12 539 patients (from 343 hospitals in 66 countries) undergoing elective or emergency gastrointestinal resection in high, middle, and low-HDI countries (GlobalSurg 2) between January and July 2016.^2^ It aimed to estimate the burden of SSI and quantify the differences in SSI rates across varying HDI settings. GlobalSurg 2 recruited consecutive patients undergoing elective or emergency or gastrointestinal resection for a prespecified 2-week period at each participating hospital. The study assessed the patients for SSIs at 30 days following the surgery in line with the US Centers for Disease Control and Prevention definition by trained SSI assessors.^2^ The dataset disaggregated surgical patients by wound contamination: clean-contaminated, contaminated, and dirty surgery. For this analysis, SSI rates for contaminated and dirty surgery were aggregated into one category (tables1  2). Limitations of the study have been included in online supplemental appendix S4.
2. *Intervention effect size*: Since our primary aim was to estimate the potential cost increases or savings from SSIs using triclosan-coated sutures, we used estimated effect sizes from a recently published meta-analysis of five high-quality randomized trials.^11^ In keeping with frequentist statistical techniques, we used the 95% CI rather than the central point estimate, replicating significance results and policy decision-making. In such methodologies, if the CI crosses one, this indicates non-significance; the central point estimate is not used in isolation. The published meta-analysis established a 95% CI of 0.74 to 1.09 (central point estimate 0.90, p=0.29).^11^ Therefore, 0.74 then acts as the lower bound (ie, reduction in SSI) and 1.09 as the upper bound (ie, increase in SSI). In the base case, we did not calculate models for the central point estimate, in keeping with this preplanned methodology.
3. *Cost of SSI*: The cost of an SSI was informed by a study that estimated the postoperative costs of patients with and without an SSI in middle-HDI countries. The study found that the proportion of postoperative hospital stay costs to total postoperative healthcare costs was 0.92.^7^ Therefore, the additional cost of an SSI was based on the cost of the hospital stay, as well as the expenses for treatments (dressings, investigations, medications, and postoperative follow-up) resulting from the SSI. The postoperative length of hospital stay for both patients with and without an SSI was sourced from the GlobalSurg 2 dataset.^2^ The hospital bed-day costs were sourced from the WHO-CHOICE country-specific costs (table 1).^20^ The bed-day costs were averaged for all countries in a given HDI category, and for the country-level analysis, each country was assigned a specific country-level cost from the WHO-CHOICE country-specific costs. All costs were converted to US dollars using purchasing power parity (PPP) exchange rates, and where the PPP conversion rates were not available, the implied PPP conversion rates were used.^21 22^ The costs were adjusted to 2022 US dollars using the Consumer Price Index (CPI) published by the World Bank.^23^ Where the CPI rates were not available, the rates were estimated by taking an average of countries in the same HDI category in each region: Arab, East Asia and the Pacific, Europe, Europe and Central Asia, Latin America and the Caribbean, North America, South Asia, and Sub-Saharan Africa.
4. *Suture cost*: The costs of plus antibacterial (triclosan-coated) and uncoated sutures were sourced from a systematic review and model-based economic evaluation that estimated the costs and outcomes of using the two types of sutures among abdominal surgery patients in the UK.^15^ The sutures are manufactured by Ethicon, J&J MedTech, and the costs were assumed to be the cost in all countries as this reflects the cost of procuring the suture from the manufacturer of the product. After converting the costs to 2022 US dollars, it was estimated that coated suture cost $35 and uncoated suture cost $28.^21 23^

**Table 1:** Model input parameters

Patient category	Baseline SSI rate (%)	Intervention arm SSI rate (%)	Length of hospital stay (patients with SSI)	Length of hospital stay (patients without SSI)	Hospital bed-day cost (all patients)	Cost of hospital stay (patients with SSI)	Cost of hospital stay (patients without SSI)
High-HDI countries: clean-contaminated wounds	7.6	6.8	11.40	4.20	$1610	$18 357	$6763
High-HDI countries: contaminated-dirty wounds	19.0	17.1	14.20	6.60	$1610	$22 866	$10 628
Middle-HDI countries: clean-contaminated wounds	11.1	10.0	6.20	2.90	$138	$856	$401
Middle-HDI countries: contaminated-dirty wounds	20.0	18.0	11.50	5.50	$138	$1588	$760
Low-HDI countries: clean-contaminated wounds	16.1	14.5	7.80	4.30	$92	$720	$397
Low-HDI countries: contaminated-dirty wounds	39.0	35.1	15.20	8.20	$92	$1403	$757

Baseline SSI rate data were sourced from the GlobalSurg 2 dataset,^2^ which includes 12 539 patients across 343 hospitals in 66 countries. LoS refers to the average length of hospital stay (in days). The LoS data were obtained from GlobalSurg 2.^2^ Bed-day cost data were extracted from WHO-CHOICE estimates.^20^ Data for all wound types are not included, as the source did not provide aggregated wound data.

HDI, Human Development Index; SSI, surgical site infection.

**Table 2:** Sources of the data and possible uncertainties surrounding the data

Item	Data source short name	Possible variations between countries	Possible variations within countries
Baseline SSI rates	GlobalSurg 2^2^	Used country-specific data. Low chances of bias because of using country-specific data from a prospective international study.	Model used country averages, but SSI rates may vary within a country.
Effect size	Ademuyiwa *et al*^11^	Effect sizes may vary by country due to epidemiological factors, but a global effect size was used being the best available data.	Effect size may vary within a country, but this was the best available data.
Coated and uncoated suture costs	Edwards *et al*^15^	Cost of the suture was available from only one supplier, but the actual cost may vary by country. Wide range of the costs used in the sensitivity analysis.	There might be within-country variations that cannot be captured with the available data.
Ratio of postoperative hospital costs to all healthcare costs	Monahan *et al*^7^	The ratio may vary by country, but this was the best available data.	The ratio may vary within a country, but this was the best available data.
Length of hospital stay (LoS)	GlobalSurg 2^2^	Used HDI group averages assuming that the LoS will apply to all countries in a given HDI category.	The LoS may vary within a country, but this was the best available data.
Bed-day costs	WHO-CHOICE estimates^20^	Used country-specific data. Low chances of bias because of using country-specific data from a prospective international study.	Model used country averages, but SSI rates may vary within a country.

HDI, Human Development Index; SSI, surgical site infection.

### Outcome measures

This analysis estimated the preoperative and postoperative costs of patients with SSI within 30 days of an operation as defined by the US Centers for Disease Control and Prevention.^24^ SSI rates for coated and uncoated sutures were estimated to quantify and value the postoperative costs associated with each strategy. The timeframe for this evaluation was 30 days, in line with the timeframe for assessing SSIs used in the trial from which SSI rate estimates were sourced.^2^ The costs and effectiveness of the strategies were not discounted because the model timeframe was less than 1 year.^25^

### Statistical analysis

To perform the modeling activity, several assumptions were made: (1) the only difference in pre, and intraoperative resource use and total costs between the arms was the cost of sutures, so the costs of the initial operation (other than the fascial suture cost) and postoperative care (other than the management of SSI) were not included in the model; (2) effect size of the triclosan-coated suture versus uncoated suture from the meta-analysis was applicable across all HDI countries because the meta-analysis pooled effectiveness data across country HDI categories^11^; and (3) uncoated suture was the standard practice in the hospitals that participated in the GlobalSurg 2 study.^2^ As such, the baseline rates reported in the study represented SSI rates for the uncoated suture arm. Bed-day unit costs were not available in eight countries, so they were estimated by taking the averages of countries in the same HDI category in the same region. Similarly, baseline SSI rates for 11 countries were not available and were imputed by taking averages of countries in the same HDI category in the same region. 26 countries had missing CPI data for some years and were also imputed by taking the averages of countries in the same HDI category in their respective regions (full list in online supplemental appendix S1).

We estimated the probability of each patient suffering an SSI after surgery and the expected cost of the SSI informed by the probability of suffering the SSI. However, we applied the 100% cost of suture to the patient in each arm as suture was used on the patient regardless of SSI status. The primary analysis estimated the difference in average total healthcare costs associated with an SSI between triclosan-coated suture and uncoated suture surgery in high, middle, and low-HDI countries separately. The analysis estimated the range of differences in costs associated with SSIs between the two groups based on the range of the effect size of the intervention. The lower and upper bounds of effect size from the meta-analysis (0.74, 1.09) were multiplied by the baseline SSI rates in each country and HDI categories to estimate the SSI rates in the intervention arm for each country and HDI categories.^11^ The absolute risk difference was estimated as the difference in SSI rates between the arms (uncoated minus coated suture arms). The cost difference per patient was estimated as the product of the absolute risk difference in SSIs and the average cost of an SSI plus the absolute difference in the cost of suture. The analysis conducted for the HDI categories was repeated for the country-level analysis to obtain estimates for the difference in costs associated with an SSI per patient by country as subgroup analyses.

The average cost difference per patient (CD) with coated suture versus uncoated suture was calculated as:


CD=(CC+RCS×CS)−(CU+RUS×CS)

CS=((CBD/PHH)×LoS⁡SSI)−(CBD×LoS⁡ no SSI)

where CC is cost of coated suture, CU is cost of uncoated suture, RCS is SSI rate with coated suture, RUS is SSI rate with uncoated suture, CS is treatment cost per SSI, CBD is cost of bed-day, PHH is the proportion of hospital stay costs to all postoperative healthcare costs, LoS SSI is the average length of hospital stay for a patient with an SSI, and LoS no SSI is the average length of hospital stay for a patient without an SSI.

Subgroup analyses were performed to understand total costs by wound contamination group (ie, clean-contaminated or contaminated-dirty) to establish the effect of wound contamination in HDI stratified countries. The analysis was then repeated for each individual of the 193 countries on the HDI list based on specific healthcare-related costs.

Deterministic sensitivity analysis (DSA) was conducted to assess the sensitivity of the results to changes in the model input parameters. DSA assessed the impact of variation in suture cost, hospital stay costs, and the effect size. DSA involves varying one parameter while holding the rest of the parameters constant to assess the impact of the parameter being varied on the results.^25^ The following DSAs were conducted: (1) the costs of coated and uncoated sutures were varied upward and downward by 50%; (2) the estimated proportion of hospital stay costs to all postoperative healthcare costs was varied upward and downward based on the lower and upper bounds of the 95% CI (0.88 to 0.96) of this proportion; (3) threshold analysis was conducted to establish the effect size that would make the cost difference zero; and (4) best-case and worst-case scenario analyses were conducted. In the best-case scenario, the upper bound for the cost of uncoated suture ($42) was used, along with the lower bounds of the effect size (0.74), the cost of coated suture ($18) and the proportion of hospital stay costs to all healthcare costs (0.88), as the higher proportion reflects the lower cost of SSI. The worst-case scenario used the reciprocal bounds of those applied in the best-case scenario: the cost of uncoated suture ($14), effect size (1.09), cost of coated suture ($53), and the proportion of hospital stay costs to all postoperative healthcare costs (0.96). The primary analysis adopted a fully pooled multicountry costing approach that pooled both effectiveness data from all countries in a given HDI category and aggregated cost data from all the countries in a given HDI category.^26^ The subgroup analyses conducted at the country level applied country-specific effectiveness and cost estimates. All analyses were conducted in Microsoft Excel for Microsoft 365 V.2405.

## Results

### Baseline data

For clean-contaminated wounds, the average total cost difference between patients with and without an SSI was $11 594 in high-HDI countries, $456 in middle-HDI countries, and $323 in low-HDI countries. For contaminated-dirty wounds, the average total cost difference between patients with and without an SSI was $12 238 in high-HDI countries, $829 in middle-HDI countries, and $646 in low-HDI countries (table 3).

**Table 3:** Comparative costs of an SSI in the triclosan-coated and uncoated suture groups (with effect size ranging from 0.74 to 1.09)

Patient category	Average total cost difference for patients with and without an SSI	Coated suture*	Uncoated suture	Cost difference per patient†
High-HDI countries: all wounds	$11 916	$1382 to $2019	$1848	−$466 to $171
High-HDI countries: clean-contaminated wounds	$11 594	$776 to $1026	$1028	−$253 to $97
High-HDI countries: contaminated-dirty wounds	$12 238	$2032 to $2726	$2726	−$694 to $250
Middle-HDI countries: all wounds	$642	$120 to $161	$144	−$23 to $18
Middle-HDI countries: clean-contaminated wounds	$456	$78 to $99	$86	−$8 to $13
Middle-HDI countries: contaminated-dirty wounds	$829	$177 to $244	$221	−$43 to $25
Low-HDI countries: all wounds	$484	$152 to $208	$186	−$34 to $22
Low-HDI countries: clean-contaminated wounds	$323	$81 to $103	$90	−$9 to $13
Low-HDI countries: contaminated-dirty wounds	$646	$256 to $361	$326	−$71 to $34

* The first value was estimated at 0.74 effect size, while the second value was estimated at 1.09 effect size.

† Negative is cost saving due to use of coated suture; positive is cost increase.

HDI, Human Development Index; SSI, surgical site infection.

### Primary analysis

When all wounds were included in the model (clean-contaminated and contaminated-dirty), the average SSI-related cost per patient with coated suture in high-HDI countries ranged from $1382 to $2019 compared with $1848 for uncoated suture. As such, coated suture was potentially associated with reduced costs by $466 or increased costs by $171 (table 3). Similarly, in middle-HDI countries, the mean SSI-related cost of coated suture ranged from $120 to $161 compared with $144 for uncoated suture, such that coated suture either reduced the costs by $23 or increased the costs by $18. In low-HDI countries, the average SSI-related cost of coated suture ranged from $152 to $208 compared with $186 for uncoated suture, which potentially reduced the average costs by $34 or increased the costs by $22. Similar results were observed when the model was run using the 0.90-point estimate of the effect size (online supplemental appendix S2). When all wounds were included in the model, coated suture was associated with reductions of $175, $4, and $8 in average costs in high, middle, and low HDIs, respectively (online supplemental table S1).

### Subgroup analysis: wound categories

**Figure 2:**
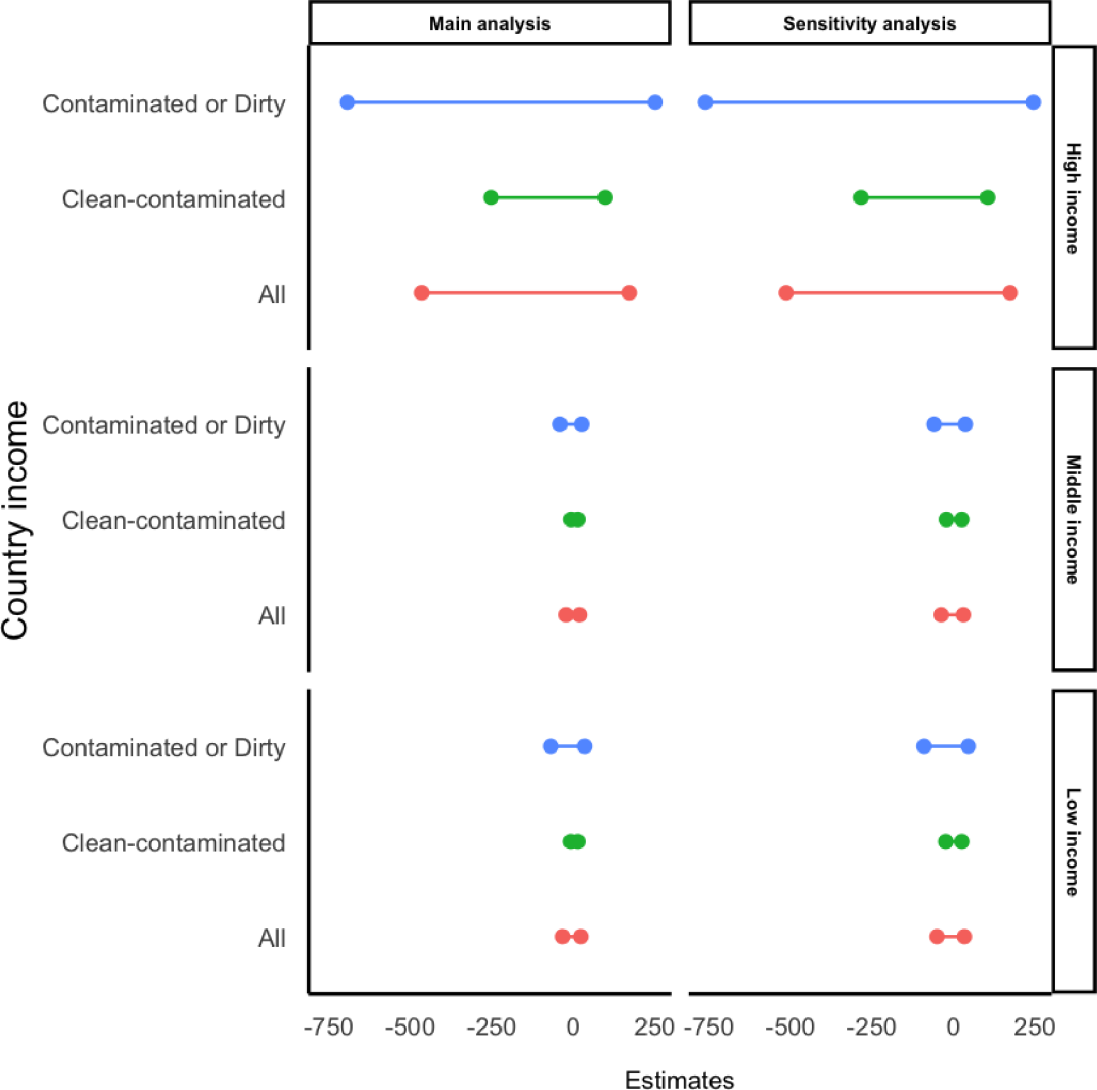
Primary and sensitivity analysis results.

Coated sutures reduced more costs in the contaminated-dirty wound category compared with the clean-contaminated wound category in all the three HDI categories (figure 2). In high-HDI countries, for example, coated suture saved up to $694 or increased the cost up to $250 in the contaminated-dirty wound category compared with the clean-contaminated wound category, where it saved up to $253 or increased the cost up to $97 (table 3).

### Country-level analyses

When all wounds were included in the model, the results were similar to the primary analysis in 184 of the 193 countries where coated suture could reduce or increase the SSI-related costs (online supplemental table S2). However, in nine countries, coated suture was associated with increased costs even when the effect size was 0.74. Higher cost savings were observed in high-HDI countries (online supplemental figure S2 and S3). For example, at the effect size of 0.74, the three biggest savings were observed in Brunei Darussalam ($3389), Lebanon ($2833), and Ireland ($2386). At 0.74 effect size, the three biggest increases in costs were observed in Madagascar ($3.30), Nepal ($2.87), and Guinea ($2.46) (online supplemental table S2). Similar results were observed for the clean-contaminated and contaminated-dirty subgroup analyses. In the clean-contaminated model, coated suture was associated with cost increases in 15 countries compared with seven countries in the contaminated-dirty model (online supplemental tables S3 and S4).

### Sensitivity analysis results

The results by country HDI categories were sensitive to the effect size (online supplemental figure S1, S2 and S4) and sensitive when the cost of either coated or uncoated suture was reduced or increased by 50% for clean-contaminated dirty model (online supplemental appendix S3, online supplemental tables S5–S8). When the proportion of hospital stay costs to overall postoperative healthcare costs was adjusted downward and upward, the results were similar to the primary analysis (online supplemental tables S9 and S10). In all the changes to the suture costs or proportional hospital stay costs to all postoperative healthcare costs, coated suture could reduce or increase the costs associated with an SSI (table 4). The effect size that would make the cost difference zero was 1.00, 0.92, and 0.93 in high, middle, and low-HDI countries, respectively. In the best-case scenario, coated suture was associated with cost savings in all country HDI categories (online supplemental table S11). In the worst-case scenario, coated suture was associated with an increase in costs across all country HDI categories (online supplemental table S12).

**Table 4:** Summary of sensitivity analysis results

Patient category	Cost of coated suture was increased by 50%	Cost of coated suture was decreased by 50%	Cost of uncoated suture was increased by 50%	Cost of uncoated suture was decreased by 50%	The proportion of hospital stay costs to all healthcare costs was adjusted to 0.88	The proportion of hospital stay costs to all healthcare costs was adjusted to 0.96	Best-case scenario	Worst-case scenario
High income: all wounds	−$448 to $189	−$483 to $155	−$480 to $157	−$452 to $185	−$500 to +$183	−$430 to +$159	−$533	$192
High income: clean-contaminated wounds	−$235 to $115	−$270 to $80	−$267 to $83	−$239 to $111	−$270 to +$104	−$234 to +$91	−$303	$124
High income: contaminated-dirty wounds	−$676 to $268	−$711 to $233	−$708 to $236	−$680 to $264	−$749 to +$269	−$637 to +$230	−$783	$265
Middle income: all wounds	−$5 to $36	−$40 to $1	−$37 to $3	−$9 to $31	−$25 to +$19	−$20 to +$17	−$57	$49
Middle income: clean-contaminated wounds	$10 to $31	$25 to −$4	−$22 to −$2	$6 to $26	−$9 to +$13	−$6 to +$12	−$41	$44
Middle income: contaminated-dirty wounds	−$25 to $43	−$60 to $8	−$57 to $10	−$29 to $38	−$46 to +$26	−$38 to +$23	−$78	$55
Low income: all wounds	−$16 to $40	−$51 to $5	−$48 to $7	−$20 to $35	−$37 to +$23	−$30 to +$20	−$69	$52
Low income: clean-contaminated wounds	$9 to $31	−$26 to −$4	−$23 to −$1	$5 to $27	−$10 to +$13	−$7 to +$12	−$42	$44
Low income: contaminated-dirty wounds	−$52 to $52	−$87 to $17	−$85 to $20	−$57 to $48	−$77 to +$37	−$63 to +$32	−$109	$64

The first value was estimated at 0.74 effect size, while the second value was estimated at 1.09 effect size.

## Discussion

This study has demonstrated that, across health systems globally, uncertainty exists around potential cost savings when using triclosan-coated sutures to reduce SSI. These findings highlight the economic uncertainty, influenced by factors such as baseline SSI rates, wound contamination levels, and healthcare systems. While cost savings were more pronounced in contaminated-dirty wounds and high-HDI countries, uncertainties in the intervention’s effectiveness, reflected in a wide CI for its effect size, highlight the need for caution in policy and procurement decisions. This study does not justify routine use of triclosan-coated suture across all patients and whole health systems.

The subgroup and sensitivity analyses provide the necessary deeper insights into the variability and robustness of the results to justify the conclusions. Subgroup analysis revealed that cost savings were more significant in patients with contaminated-dirty wounds, highlighting the differential economic impact based on wound contamination levels. Sensitivity analysis demonstrated the stability of the findings under varying cost inputs and assumptions, identifying key parameters influencing cost differences. The main results were observed in 184 of 193 countries included in the country-level secondary analyses. However, in nine countries, coated suture was associated with an increase in costs even at the best possible effect size of the intervention.

A key strength of this analysis, based on the CI from a published meta-analysis of high-quality randomized trials, is its reliance on robust and reliable data, which strictly minimizes bias and enhances the validity of findings. By focusing exclusively on high-quality studies, this approach ensures that the economic analysis reflects evidence from well-designed trials with greater methodological rigor. However, a limitation of this approach is the exclusion of low-quality randomized trials, which often report overwhelmingly positive effects for interventions like triclosan-coated sutures. While these studies might exaggerate benefits, their exclusion might overlook potentially favorable scenarios. Despite the debate surrounding the decision to prioritize high-quality data, this analysis deliberately adopted this approach to ensure credibility and relevance, particularly as high-quality randomized trials now numerically and geographically (including high, middle, and low-HDI settings) outweigh the low-quality data, providing a more comprehensive evidence base. This model used the best available data, including the effect size and SSI rates from a real trial conducted across HDI settings. However, there are resource use variations and SSI incidence within and between countries that are not captured by the average data used in the model. Further, the analysis was based on the same level of effectiveness of the intervention globally. However, the effectiveness may vary between and within countries based on epidemiological factors and healthcare systems. Still, a wide range of sensitivity analyses were conducted on the effectiveness of the intervention at HDI categories and country level, and the results show that the intervention can reduce or increase the average costs, except in a few scenarios. The sensitivity analysis results on the suture costs indicate the price range that will be associated with cost increases or reductions. This range gives an indication to the industry on the feasible suture price range but also can help governments with negotiations on the suture reimbursement costs.

Cost savings were greater in high-HDI countries due to higher relative costs and input parameters compared with low- and middle-HDI countries, where healthcare costs are generally lower.^16^ In low- and middle-HDI countries, a significant proportion of patients incur out-of-pocket expenses, which were not accounted for due to the narrower healthcare perspective of this study.^27^ A broader societal perspective might reveal greater cost savings in these settings. Interestingly, middle-HDI countries showed lower costs than low-HDI countries, likely due to shorter lengths of stay, though the reasons for this difference remain unclear.

Results from three previous evaluations suggested that triclosan-coated sutures are potentially cost saving in SSI prevention. A US study reported cost reductions between $4109 and $53 244 per SSI prevented, with higher savings when a societal perspective was used over a hospital or third-party perspective. Further, higher cost savings were noted when the likelihood of preventing SSIs increased.^13^ Similarly, a 2019 Italian hospital analysis estimated an annual savings of €14 785 ($23 204 converted to 2022 US dollars in the current study).^12^ An Egyptian hospital study projected an annual savings of $1 517 727 when using coated sutures for various surgeries.^14^ Also, a UK study estimated that triclosan-coated sutures were associated with $22 cost savings per patient and observed higher savings in non-clean wounds compared with clean wounds.^15^ The point estimate of the current study reports cost reductions of $175, $4, and $8 when all wound categories are included in the model for high, middle, and low HDIs, respectively. While these studies align with the cost-saving potential of coated sutures, their findings differ from the current analysis, as they rely on central point estimate effect sizes rather than a CI-based approach that includes only high-quality trials.

This economic analysis is strengthened by its reliance on high-quality randomized trial data and global SSI baseline estimates, allowing for robust and comprehensive cost evaluations across 193 countries. These findings provide valuable insights for decision-makers at the country level. However, a key limitation lies in the CI of the effect size, which indicates that coated sutures may reduce or increase SSIs. This introduces uncertainty, particularly when the effect size exceeds 1, identifying the need for cautious interpretation of the results.

Future research should address the significant uncertainty surrounding the clinical effectiveness and economic impact of triclosan-coated sutures for SSI prevention in abdominal surgery. Further randomized trials should adhere to rigorous methodologies, as outlined in the recent systematic review and meta-analysis, to provide more definitive evidence on the effectiveness of triclosan-coated sutures compared with uncoated sutures.^28^ This study illustrates how economic analyses can successfully be based on clinical CIs, which can aid clinical policy making.

## Supplementary material

10.1136/bmjsit-2025-000383 online supplemental file 1

## Data Availability

All data relevant to the study are included in the article or uploaded as supplementary information.
