# Supplementary material for: Economic analysis of triclosan-coated versus uncoated sutures at preventing surgical site infection in patients undergoing abdominal surgery
Source: BMJ Surg Interv Health Technol. 2025 Jun 12;7(1):e000383. doi: 10.1136/bmjsit-2025-000383 (PMC12164619; doi:10.1136/bmjsit-2025-000383)
Supplement: online supplemental file 1 [file bmjsit-7-1-s001.docx]

**An economic analysis of triclosan coated versus uncoated sutures at preventing surgical site infection in patients undergoing abdominal surgery**

**Supplementary file**

**Contents**

[**Appendix S1: Methods** 2](#_Toc193721977)

[**Appendix S2: Results - cost-savings associated with coated suture** 3](#_Toc193721978)

[**Appendix S3: Sensitivity analysis results** 19](#_Toc193721979)

[**Appendix S4: Discussion** 28](#_Toc193721980)

# **Appendix S1: Methods**

**Missing data**

Bed-day unit costs were not available from Democratic Republic of the Congo, Hong Kong, China (SAR), Liechtenstein, Palestine, Somalia, South Sudan, and Zimbabwe so they were estimated by taking the averages of countries in the same Human Development Index (HDI) category in the same region. The regions were defined as Arab, East Asia and the Pacific, Europe, Europe and Central Asia, Latin America and the Caribbean, North America, South Asia, and Sub-Saharan Africa. Similarly, baseline SSI rates were not available from Chad, Central African Republic, Democratic Republic of the Congo, Eritrea, Hong Kong, China (SAR), Marshall Islands, Nauru, Niger, San Marino and Tuvalu which were imputed by taking averages of countries in the same HDI category in the same region as the country of interest. Afghanistan, Bosnia and Herzegovina, Comoros, Liberia, Myanmar, Russian Federation, San Marino Swaziland, Syrian Arab Republic, Tajikistan, Venezuela, Yemen, Uzbekistan, Tuvalu, Nauru, Barbados, Democratic, Republic of the Congo, Kiribati, Seychelles, Timor-Leste, Marshall Islands, Eritrea, Cuba, Argentina, Andorra, and Palestine had missing CPI data for some years and were also imputed by taking the averages of countries of countries in the same HDI category in their respective regions.

# **Appendix S2: Results - cost-savings associated with coated suture**

This study used a frequentist statistical approach of data analysis that focuses on presenting 95% confidence interval (CI) of results rather than the point estimates. However, we have included results of the point estimates observed when the effect size of the intervention was 0.90 odds ratio as reported of a meta-analysis of high-quality trials for coated suture. The point estimates presented in Table S1 below have been included for comparative purposes and not as a key result per se.

Table S1: Point estimates presenting average difference in costs associated with an SSI by HDI categories

| **HDI group** | **Patient category** | **Coated suture group** | **Uncoated suture group** | **Cost difference per patient** |
| --- | --- | --- | --- | --- |
| High HDI | All wounds | $1,673 | $1,848 | -$175 |
|  | Clean-contaminated wounds | $936 | $1,028 | -$93 |
|  | Contaminated-dirty wounds | $2,463 | $2,726 | -$262 |
| Middle HDI | All wounds | $140 | $144 | -$4 |
|  | Clean-contaminated wounds | $88 | $86 | $2 |
|  | Contaminated-dirty wounds | $209 | $221 | -$12 |
| Low HDI | All wounds | $178 | $186 | -$8 |
|  | Clean-contaminated wounds | $91 | $90 | $1 |
|  | Contaminated-dirty wounds | $304 | $326 | -$22 |

Table S2: Country-level average difference in costs associated with an SSI (all wounds)

| **No** | **Country** | **Bed day cost** | **Cost difference per patient: lower bound** | **Cost difference per patient: Upper bound** |
| --- | --- | --- | --- | --- |
| **Estimated using complete original data** | | | | |
| 1 | Switzerland | $3,950.07 | -$1,453.26 | $513.03 |
| 2 | Norway | $2,781.40 | -$1,028.86 | $366.12 |
| 3 | Iceland | $1,023.29 | -$353.19 | $132.24 |
| 4 | Denmark | $2,542.65 | -$929.30 | $331.66 |
| 5 | Sweden | $5,330.14 | -$1,884.72 | $662.38 |
| 6 | Germany | $4,337.52 | -$1,578.63 | $556.43 |
| 7 | Ireland | $6,571.18 | -$2,386.44 | $836.05 |
| 8 | Singapore | $2,587.59 | -$921.35 | $328.91 |
| 9 | Australia | $1,786.37 | -$655.65 | $236.93 |
| 10 | Netherlands | $6,273.92 | -$2,295.27 | $804.49 |
| 11 | Belgium | $3,111.58 | -$1,069.35 | $380.14 |
| 12 | Finland | $3,987.51 | -$1,357.72 | $479.96 |
| 13 | United Kingdom | $2,066.10 | -$723.34 | $260.36 |
| 14 | New Zealand | $1,637.46 | -$582.50 | $211.61 |
| 15 | United Arab Emirates | $4,422.02 | -$1,428.29 | $504.39 |
| 16 | Canada | $2,803.29 | -$1,006.25 | $358.29 |
| 17 | Republic of Korea | $872.36 | -$298.88 | $113.44 |
| 18 | United States of America | $4,417.92 | -$1,596.04 | $562.45 |
| 19 | Luxembourg | $5,187.65 | -$1,800.82 | $633.34 |
| 20 | Austria | $3,623.75 | -$1,237.61 | $438.38 |
| 21 | Slovenia | $2,286.57 | -$772.64 | $277.43 |
| 22 | Japan | $2,088.32 | -$717.86 | $258.47 |
| 23 | Israel | $763.20 | -$259.58 | $99.83 |
| 24 | Malta | $1,927.17 | -$623.26 | $225.72 |
| 25 | Spain | $2,618.17 | -$882.65 | $315.51 |
| 26 | France | $2,149.16 | -$733.63 | $263.93 |
| 27 | Cyprus | $1,879.73 | -$618.89 | $224.21 |
| 28 | Italy | $3,146.96 | -$1,058.71 | $376.45 |
| 29 | Estonia | $2,150.94 | -$713.94 | $257.11 |
| 30 | Czech Republic | $4,438.61 | -$1,491.16 | $526.15 |
| 31 | Greece | $987.92 | -$325.01 | $122.48 |
| 32 | Bahrain | $614.11 | -$190.14 | $75.79 |
| 33 | Poland | $1,837.04 | -$597.21 | $216.70 |
| 34 | Latvia | $4,349.61 | -$1,388.92 | $490.76 |
| 35 | Lithuania | $4,310.06 | -$1,407.05 | $497.03 |
| 36 | Croatia | $3,134.20 | -$996.80 | $355.02 |
| 37 | Qatar | $3,398.51 | -$1,121.36 | $398.14 |
| 38 | Saudi Arabia | $186.15 | -$53.34 | $28.44 |
| 39 | Portugal | $2,455.38 | -$785.95 | $282.03 |
| 40 | Chile | $2,480.54 | -$795.97 | $285.51 |
| 41 | Slovakia | $5,254.61 | -$1,727.16 | $607.84 |
| 42 | Turkey | $2,091.02 | -$661.25 | $238.87 |
| 43 | Hungary | $1,015.99 | -$320.12 | $120.79 |
| 44 | Kuwait | $598.98 | -$184.15 | $73.72 |
| 45 | Montenegro | $3,988.67 | -$1,266.07 | $448.23 |
| 46 | Saint Kitts and Nevis | $873.99 | -$275.73 | $105.42 |
| 47 | Uruguay | $290.26 | -$84.65 | $39.28 |
| 48 | Romania | $517.19 | -$156.80 | $64.25 |
| 49 | Antigua and Barbuda | $708.80 | -$216.77 | $85.01 |
| 50 | Brunei Darussalam | $10,161.17 | -$3,389.12 | $1,183.13 |
| 51 | Bahamas | $2,227.07 | -$698.30 | $251.70 |
| 52 | Panama | $170.08 | -$46.36 | $26.02 |
| 53 | Oman | $272.74 | -$79.29 | $37.42 |
| 54 | Georgia | $107.58 | -$27.33 | $19.44 |
| 55 | Trinidad and Tobago | $245.54 | -$70.34 | $34.32 |
| 56 | Malaysia | $492.72 | -$148.37 | $61.34 |
| 57 | Costa Rica | $249.17 | -$71.84 | $34.84 |
| 58 | Serbia | $318.17 | -$93.42 | $42.31 |
| 59 | Thailand | $233.36 | -$74.53 | $35.78 |
| 60 | Kazakhstan | $373.45 | -$110.84 | $48.34 |
| 61 | Belarus | $412.70 | -$124.14 | $52.95 |
| 62 | Bulgaria | $1,332.51 | -$413.90 | $153.25 |
| 63 | Palau | $2,353.89 | -$736.72 | $264.99 |
| 64 | Mauritius | $828.24 | -$254.54 | $98.09 |
| 65 | Grenada | $112.66 | -$29.33 | $20.13 |
| 66 | Albania | $224.42 | -$67.69 | $33.41 |
| 67 | China | $61.55 | -$13.77 | $14.74 |
| 68 | Armenia | $77.24 | -$18.57 | $16.40 |
| 69 | Mexico | $472.20 | -$144.28 | $59.92 |
| 70 | Iran (Islamic Republic of) | $997.87 | -$310.49 | $117.45 |
| 71 | Sri Lanka | $511.15 | -$156.40 | $64.12 |
| 72 | Saint Vincent and the Grenadines | $1,153.34 | -$413.60 | $153.14 |
| 73 | Dominica | $470.26 | -$158.92 | $64.99 |
| 74 | Ecuador | $120.43 | -$33.54 | $21.59 |
| 75 | North Macedonia | $1,537.40 | -$497.84 | $182.31 |
| 76 | Republic of Moldova | $139.31 | -$47.97 | $26.58 |
| 77 | Peru | $732.72 | -$236.58 | $91.87 |
| 78 | Maldives | $42.16 | -$8.85 | $13.04 |
| 79 | Azerbaijan | $344.03 | -$104.40 | $46.12 |
| 80 | Brazil | $59.00 | -$11.59 | $13.99 |
| 81 | Colombia | $600.84 | -$210.11 | $82.71 |
| 82 | Libya | $440.71 | -$149.64 | $61.77 |
| 83 | Algeria | $265.94 | -$80.74 | $37.92 |
| 84 | Turkmenistan | $334.19 | -$128.32 | $54.39 |
| 85 | Guyana | $55.56 | -$19.71 | $16.80 |
| 86 | Mongolia | $73.74 | -$18.30 | $16.31 |
| 87 | Dominican Republic | $181.17 | -$61.12 | $31.13 |
| 88 | Tonga | $174.76 | -$57.49 | $29.88 |
| 89 | Jordan | $64.12 | -$13.58 | $14.68 |
| 90 | Ukraine | $188.69 | -$54.86 | $28.97 |
| 91 | Tunisia | $335.59 | -$113.11 | $49.13 |
| 92 | Paraguay | $162.08 | -$60.67 | $30.98 |
| 93 | Fiji | $70.45 | -$16.99 | $15.86 |
| 94 | Egypt | $99.77 | -$32.67 | $21.29 |
| 95 | Vietnam | $12.33 | $1.85 | $9.34 |
| 96 | Saint Lucia | $320.79 | -$102.32 | $45.40 |
| 97 | Lebanon | $8,942.04 | -$2,832.80 | $990.56 |
| 98 | South Africa | $254.24 | -$105.96 | $46.65 |
| 99 | Indonesia | $43.23 | -$10.54 | $13.63 |
| 100 | Philippines | $72.86 | -$24.29 | $18.39 |
| 101 | Botswana | $371.28 | -$138.66 | $57.97 |
| 102 | Jamaica | $81.87 | -$22.73 | $17.84 |
| 103 | Samoa | $728.76 | -$276.40 | $105.65 |
| 104 | Kyrgyzstan | $43.14 | -$12.57 | $14.33 |
| 105 | Belize | $691.00 | -$251.55 | $97.05 |
| 106 | Bolivia | $40.90 | -$4.49 | $11.53 |
| 107 | Morocco | $394.84 | -$118.62 | $51.04 |
| 108 | Gabon | $629.35 | -$157.09 | $64.35 |
| 109 | Suriname | $819.89 | -$191.87 | $76.39 |
| 110 | Bhutan | $57.59 | -$12.57 | $14.33 |
| 111 | El Salvador | $532.16 | -$141.79 | $59.06 |
| 112 | Iraq | $77.56 | -$15.75 | $15.43 |
| 113 | Bangladesh | $15.70 | $1.12 | $9.59 |
| 114 | Nicaragua | $82.46 | -$18.55 | $16.40 |
| 115 | Cabo Verde | $180.51 | -$47.20 | $26.32 |
| 116 | Equatorial Guinea | $307.62 | -$108.89 | $47.67 |
| 117 | India | $22.76 | -$0.27 | $10.07 |
| 118 | Micronesia (Federated States of) | $67.92 | -$13.41 | $14.62 |
| 119 | Guatemala | $106.83 | -$27.64 | $19.54 |
| 120 | Honduras | $87.78 | -$22.62 | $17.81 |
| 121 | Lao People's Democratic Republic | $105.15 | -$34.10 | $21.78 |
| 122 | Vanuatu | $133.04 | -$40.76 | $24.09 |
| 123 | Sao Tome and Principe | $26.00 | -$3.31 | $11.12 |
| 124 | Namibia | $113.14 | -$29.20 | $20.08 |
| 125 | Ghana | $49.31 | -$11.78 | $14.05 |
| 126 | Kenya | $31.80 | -$5.89 | $12.02 |
| 127 | Nepal | $10.71 | $2.87 | $8.98 |
| 128 | Cambodia | $31.11 | -$5.23 | $11.79 |
| 129 | Congo | $394.68 | -$137.56 | $57.59 |
| 130 | Angola | $105.73 | -$39.43 | $23.63 |
| 131 | Cameroon | $113.24 | -$45.68 | $25.79 |
| 132 | Zambia | $19.50 | -$0.07 | $10.00 |
| 133 | Papua New Guinea | $23.90 | -$4.59 | $11.57 |
| 134 | Solomon Islands | $161.29 | -$72.51 | $35.07 |
| 135 | Haiti | $74.01 | -$32.31 | $21.16 |
| 136 | Uganda | $21.90 | -$6.49 | $12.22 |
| 137 | Nigeria | $35.52 | -$11.52 | $13.96 |
| 138 | Rwanda | $808.30 | -$499.18 | $182.77 |
| 139 | Togo | $47.33 | -$21.50 | $17.42 |
| 140 | Pakistan | $45.41 | -$15.13 | $15.21 |
| 141 | Mauritania | $36.19 | -$12.98 | $14.47 |
| 142 | Cote d'Ivoire | $88.05 | -$53.44 | $28.48 |
| 143 | United republic of Tanzania | $9.60 | $2.37 | $9.16 |
| 144 | Lesotho | $63.28 | -$30.73 | $20.61 |
| 145 | Senegal | $152.92 | -$95.82 | $43.14 |
| 146 | Sudan | $296.71 | -$185.70 | $74.26 |
| 147 | Djibouti | $33.46 | -$14.63 | $15.04 |
| 148 | Malawi | $21.27 | -$7.46 | $12.56 |
| 149 | Benin | $9.00 | $1.62 | $9.41 |
| 150 | Gambia | $46.91 | -$26.14 | $19.03 |
| 151 | Ethiopia | $38.12 | -$19.55 | $16.74 |
| 152 | Madagascar | $7.50 | $3.30 | $8.84 |
| 153 | Guinea-Bissau | $41.61 | -$23.35 | $18.06 |
| 154 | Guinea | $6.42 | $2.46 | $9.12 |
| 155 | Mozambique | $6.67 | $2.37 | $9.16 |
| 156 | Sierra Leone | $39.34 | -$22.61 | $17.80 |
| 157 | Burkina Faso | $226.82 | -$169.27 | $68.57 |
| 158 | Burundi | $12.88 | -$2.73 | $10.92 |
| 159 | Mali | $102.24 | -$68.99 | $33.86 |
| **Incorporated some imputed data** | | | | |
| 160 | Hong Kong, China (SAR) | $225.64 | -$73.28 | $35.34 |
| 161 | Liechtenstein | $2,248.49 | -$793.71 | $284.72 |
| 162 | Andorra | $3,437.08 | -$1,130.63 | $401.35 |
| 163 | San Marino | $4,149.02 | -$1,406.71 | $496.91 |
| 164 | Argentina | $459.64 | -$142.20 | $59.20 |
| 165 | Russian Federation | $562.80 | -$171.73 | $69.42 |
| 166 | Barbados | $467.48 | -$140.52 | $58.62 |
| 167 | Seychelles | $1,377.16 | -$428.37 | $158.26 |
| 168 | Bosnia and Herzegovina | $1,209.34 | -$401.54 | $148.97 |
| 169 | Cuba | $86.87 | -$20.15 | $16.95 |
| 170 | Marshall Islands | $71.31 | -$19.16 | $16.61 |
| 171 | Uzbekistan | $62.87 | -$19.62 | $16.77 |
| 172 | Palestine, State of | $2,197.70 | -$926.47 | $330.68 |
| 173 | Venezuela | $110.19 | -$14.97 | $15.16 |
| 174 | Nauru | $72.02 | -$19.82 | $16.84 |
| 175 | Tajikistan | $43.93 | -$7.20 | $12.47 |
| 176 | Tuvalu | $62.99 | -$16.41 | $15.66 |
| 177 | Kiribati | $72.95 | -$19.77 | $16.82 |
| 178 | Swaziland | $163.96 | -$66.27 | $32.92 |
| 179 | Myanmar | $20.62 | -$1.59 | $10.53 |
| 180 | Comoros | $37.05 | -$11.45 | $13.94 |
| 181 | Timor-Leste | $26.32 | -$1.85 | $10.62 |
| 182 | Syrian Arab Republic | $98.28 | -$27.67 | $19.56 |
| 183 | Zimbabwe | $411.61 | -$221.36 | $86.60 |
| 184 | Eritrea | $13.02 | -$1.35 | $10.44 |
| 185 | Liberia | $9.11 | $0.75 | $9.72 |
| 186 | Democratic Republic of the Congo | $24.41 | -$10.25 | $13.52 |
| 187 | Afghanistan | $44.37 | -$22.90 | $17.90 |
| 188 | Yemen | $182.07 | -$100.90 | $44.90 |
| 189 | Chad | $39.05 | -$18.87 | $16.51 |
| 190 | Niger | $27.23 | -$10.91 | $13.75 |
| 191 | Central African Republic | $26.62 | -$10.51 | $13.61 |
| 192 | South Sudan | $64.90 | -$35.87 | $22.39 |
| 193 | Somalia | $190.07 | -$107.22 | $47.09 |

At the lower bound of the effect size (0.74), coated suture was associated with increased costs in Bangladesh, Benin, Guinea, Liberia, Madagascar, Mozambique, Nepal, United republic of Tanzania, and Vietnam. In 184 it could reduce the costs or increase the costs. At the upper bound (1.09), coated suture was associated with increased costs in all countries.

Table S3: Country-level average difference in costs associated with an SSI (clean-contaminated wounds).

| **No** | **Country** | **Cost difference per patient: lower bound** | **Cost difference per patient: Upper bound** |
| --- | --- | --- | --- |
| **Estimated using complete original data** | | | |
| 1 | Switzerland | -$896.85 | $320.42 |
| 2 | Norway | -$634.67 | $229.67 |
| 3 | Iceland | -$214.69 | $84.29 |
| 4 | Denmark | -$572.24 | $208.06 |
| 5 | Sweden | -$1,158.84 | $411.11 |
| 6 | Germany | -$973.23 | $346.86 |
| 7 | Ireland | -$1,472.09 | $519.55 |
| 8 | Singapore | -$565.70 | $205.80 |
| 9 | Australia | -$403.24 | $149.56 |
| 10 | Netherlands | -$1,416.91 | $500.45 |
| 11 | Belgium | -$654.63 | $236.58 |
| 12 | Finland | -$831.11 | $297.67 |
| 13 | United Kingdom | -$442.84 | $163.27 |
| 14 | New Zealand | -$356.75 | $133.47 |
| 15 | United Arab Emirates | -$870.66 | $311.36 |
| 16 | Canada | -$618.58 | $224.10 |
| 17 | Republic of Korea | -$181.17 | $72.69 |
| 18 | United States of America | -$983.19 | $350.31 |
| 19 | Luxembourg | -$1,105.13 | $392.52 |
| 20 | Austria | -$757.58 | $272.22 |
| 21 | Slovenia | -$471.58 | $173.22 |
| 22 | Japan | -$438.67 | $161.83 |
| 23 | Israel | -$156.92 | $64.29 |
| 24 | Malta | -$378.53 | $141.01 |
| 25 | Spain | -$538.96 | $196.54 |
| 26 | France | -$448.06 | $165.08 |
| 27 | Cyprus | -$376.40 | $140.27 |
| 28 | Italy | -$646.84 | $233.88 |
| 29 | Estonia | -$434.88 | $160.51 |
| 30 | Czech Republic | -$911.95 | $325.65 |
| 31 | Greece | -$196.47 | $77.98 |
| 32 | Bahrain | -$113.33 | $49.21 |
| 33 | Poland | -$362.75 | $135.54 |
| 34 | Latvia | -$845.92 | $302.79 |
| 35 | Lithuania | -$858.36 | $307.10 |
| 36 | Croatia | -$606.21 | $219.82 |
| 37 | Qatar | -$684.14 | $246.79 |
| 38 | Saudi Arabia | -$29.76 | $20.28 |
| 39 | Portugal | -$477.64 | $175.31 |
| 40 | Chile | -$483.85 | $177.46 |
| 41 | Slovakia | -$1,054.77 | $375.09 |
| 42 | Turkey | -$401.41 | $148.93 |
| 43 | Hungary | -$192.81 | $76.72 |
| 44 | Kuwait | -$109.63 | $47.93 |
| 45 | Montenegro | -$770.57 | $276.71 |
| 46 | Saint Kitts and Nevis | -$165.89 | $67.40 |
| 47 | Uruguay | -$48.81 | $26.87 |
| 48 | Romania | -$92.88 | $42.13 |
| 49 | Antigua and Barbuda | -$129.48 | $54.80 |
| 50 | Brunei Darussalam | -$2,074.59 | $728.10 |
| 51 | Bahamas | -$423.54 | $156.59 |
| 52 | Panama | -$25.42 | $18.78 |
| 53 | Oman | -$45.54 | $25.74 |
| 54 | Georgia | -$13.85 | $14.77 |
| 55 | Trinidad and Tobago | -$40.08 | $23.85 |
| 56 | Malaysia | -$87.73 | $40.34 |
| 57 | Costa Rica | -$41.02 | $24.18 |
| 58 | Serbia | -$54.19 | $28.73 |
| 59 | Thailand | -$43.08 | $24.89 |
| 60 | Kazakhstan | -$64.80 | $32.41 |
| 61 | Belarus | -$72.95 | $35.23 |
| 62 | Bulgaria | -$249.86 | $96.47 |
| 63 | Palau | -$446.99 | $164.70 |
| 64 | Mauritius | -$152.57 | $62.79 |
| 65 | Grenada | -$15.09 | $15.20 |
| 66 | Albania | -$38.68 | $23.36 |
| 67 | China | -$5.62 | $11.92 |
| 68 | Armenia | -$8.54 | $12.93 |
| 69 | Mexico | -$85.37 | $39.53 |
| 70 | Iran (Islamic Republic of) | -$186.88 | $74.67 |
| 71 | Sri Lanka | -$92.76 | $42.09 |
| 72 | Saint Vincent and the Grenadines | -$252.86 | $97.51 |
| 73 | Dominica | -$95.14 | $42.91 |
| 74 | Ecuador | -$17.75 | $16.12 |
| 75 | North Macedonia | -$302.21 | $114.59 |
| 76 | Republic of Moldova | -$27.10 | $19.36 |
| 77 | Peru | -$142.27 | $59.22 |
| 78 | Maldives | -$2.69 | $10.91 |
| 79 | Azerbaijan | -$61.05 | $31.11 |
| 80 | Brazil | -$4.21 | $11.44 |
| 81 | Colombia | -$126.96 | $53.93 |
| 82 | Libya | -$89.48 | $40.95 |
| 83 | Algeria | -$46.64 | $26.12 |
| 84 | Turkmenistan | -$77.34 | $36.75 |
| 85 | Guyana | -$9.88 | $13.40 |
| 86 | Mongolia | -$8.42 | $12.89 |
| 87 | Dominican Republic | -$35.09 | $22.12 |
| 88 | Tonga | -$32.78 | $21.32 |
| 89 | Jordan | -$5.45 | $11.86 |
| 90 | Ukraine | -$30.76 | $20.62 |
| 91 | Tunisia | -$66.99 | $33.17 |
| 92 | Paraguay | -$35.25 | $22.18 |
| 93 | Fiji | -$7.61 | $12.61 |
| 94 | Egypt | -$17.59 | $16.07 |
| 95 | Vietnam | $3.90 | $8.63 |
| 96 | Saint Lucia | -$60.05 | $30.76 |
| 97 | Lebanon | -$1,728.01 | $608.13 |
| 98 | South Africa | -$64.10 | $32.16 |
| 99 | Indonesia | -$3.83 | $11.30 |
| 100 | Philippines | -$12.53 | $14.31 |
| 101 | Botswana | -$83.52 | $38.89 |
| 102 | Jamaica | -$11.24 | $13.87 |
| 103 | Samoa | -$169.09 | $68.51 |
| 104 | Kyrgyzstan | -$5.25 | $11.79 |
| 105 | Belize | -$153.06 | $62.96 |
| 106 | Bolivia | -$0.12 | $10.02 |
| 107 | Morocco | -$73.26 | $35.33 |
| 108 | Gabon | -$95.42 | $43.01 |
| 109 | Suriname | -$116.29 | $50.23 |
| 110 | Bhutan | -$5.53 | $11.89 |
| 111 | El Salvador | -$86.56 | $39.94 |
| 112 | Iraq | -$7.29 | $12.50 |
| 113 | Bangladesh | $3.24 | $8.86 |
| 114 | Nicaragua | -$9.18 | $13.15 |
| 115 | Cabo Verde | -$27.30 | $19.43 |
| 116 | Equatorial Guinea | -$68.99 | $33.86 |
| 117 | India | $2.46 | $9.13 |
| 118 | Micronesia (Federated States of) | -$5.85 | $12.00 |
| 119 | Guatemala | -$15.11 | $15.21 |
| 120 | Honduras | -$12.00 | $14.13 |
| 121 | Lao People's Democratic Republic | -$20.08 | $16.93 |
| 122 | Vanuatu | -$24.01 | $18.29 |
| 123 | Sao Tome and Principe | $0.26 | $9.89 |
| 124 | Namibia | -$16.07 | $15.54 |
| 125 | Ghana | -$5.26 | $11.80 |
| 126 | Kenya | -$1.49 | $10.49 |
| 127 | Nepal | $4.36 | $8.47 |
| 128 | Cambodia | -$1.00 | $10.32 |
| 129 | Congo | -$87.37 | $40.22 |
| 130 | Angola | -$24.32 | $18.40 |
| 131 | Cameroon | -$29.01 | $20.02 |
| 132 | Zambia | $2.48 | $9.12 |
| 133 | Papua New Guinea | -$0.96 | $10.31 |
| 134 | Solomon Islands | -$48.14 | $26.64 |
| 135 | Haiti | -$20.77 | $17.17 |
| 136 | Uganda | -$2.52 | $10.85 |
| 137 | Nigeria | -$5.53 | $11.89 |
| 138 | Rwanda | -$353.29 | $132.27 |
| 139 | Togo | -$13.03 | $14.49 |
| 140 | Pakistan | -$7.77 | $12.67 |
| 141 | Mauritania | -$6.72 | $12.30 |
| 142 | Cote d'Ivoire | -$37.15 | $22.84 |
| 143 | United republic of Tanzania | $3.97 | $8.60 |
| 144 | Lesotho | -$19.47 | $16.71 |
| 145 | Senegal | -$67.65 | $33.40 |
| 146 | Sudan | -$131.54 | $55.51 |
| 147 | Djibouti | -$8.51 | $12.92 |
| 148 | Malawi | -$3.52 | $11.19 |
| 149 | Benin | $3.26 | $8.85 |
| 150 | Gambia | -$17.42 | $16.01 |
| 151 | Ethiopia | -$12.47 | $14.29 |
| 152 | Madagascar | $4.58 | $8.39 |
| 153 | Guinea-Bissau | -$15.59 | $15.37 |
| 154 | Guinea | $3.66 | $8.71 |
| 155 | Mozambique | $3.62 | $8.72 |
| 156 | Sierra Leone | -$15.27 | $15.26 |
| 157 | Burkina Faso | -$126.97 | $53.93 |
| 158 | Burundi | -$0.32 | $10.09 |
| 159 | Mali | -$49.91 | $27.25 |
| **Incorporated some imputed data** | | | |
| 160 | Hong Kong, China (SAR) | -$42.36 | $24.64 |
| 161 | Liechtenstein | -$486.56 | $178.40 |
| 162 | Andorra | -$689.65 | $248.70 |
| 163 | San Marino | -$861.43 | $308.16 |
| 164 | Argentina | -$84.11 | $39.09 |
| 165 | Russian Federation | -$102.01 | $45.29 |
| 166 | Barbados | -$82.92 | $38.68 |
| 167 | Seychelles | -$258.75 | $99.54 |
| 168 | Bosnia and Herzegovina | -$243.78 | $94.36 |
| 169 | Cuba | -$9.43 | $13.24 |
| 170 | Marshall Islands | -$9.07 | $13.12 |
| 171 | Uzbekistan | -$9.57 | $13.29 |
| 172 | Palestine, State of | -$578.49 | $210.22 |
| 173 | Venezuela | -$6.27 | $12.15 |
| 174 | Nauru | -$10.53 | $13.62 |
| 175 | Tajikistan | -$2.00 | $10.67 |
| 176 | Tuvalu | -$8.28 | $12.84 |
| 177 | Kiribati | -$10.40 | $13.58 |
| 178 | Swaziland | -$42.66 | $24.74 |
| 179 | Myanmar | $1.33 | $9.52 |
| 180 | Comoros | -$5.79 | $11.98 |
| 181 | Timor-Leste | $1.40 | $9.49 |
| 182 | Syrian Arab Republic | -$15.42 | $15.31 |
| 183 | Zimbabwe | -$150.55 | $62.09 |
| 184 | Eritrea | $1.02 | $9.62 |
| 185 | Liberia | $2.45 | $9.13 |
| 186 | Democratic Republic of the Congo | -$5.70 | $11.95 |
| 187 | Afghanistan | -$14.70 | $15.07 |
| 188 | Yemen | -$68.64 | $33.74 |
| 189 | Chad | -$11.77 | $14.05 |
| 190 | Niger | -$5.96 | $12.04 |
| 191 | Central African Republic | -$5.66 | $11.94 |
| 192 | South Sudan | -$23.95 | $18.27 |
| 193 | Somalia | -$73.46 | $35.40 |

At the lower bound of the effect size (0.74), coated suture was associated with increased costs in Bangladesh, Benin, Eritrea, Guinea, India, Liberia, Madagascar, Mozambique, Myanmar, Nepal, United republic of Tanzania, Timor-Leste, Sao Tome and Principe, Vietnam, and Zambia. In 178 it could reduce the costs or increase the costs. At the upper bound (1.09), coated suture was associated with an increase in costs in all countries.

Table S4: Country-level average difference in costs associated with an SSI (contaminated-dirty wounds).

| **No** | **Country** | **Cost difference per patient: lower bound** | **Cost difference per patient: Upper bound** |
| --- | --- | --- | --- |
| **Estimated using complete original data** | | | |
| 1 | Switzerland | -$1,731.47 | $609.33 |
| 2 | Norway | -$1,225.96 | $434.35 |
| 3 | Iceland | -$422.44 | $156.21 |
| 4 | Denmark | -$1,107.83 | $393.46 |
| 5 | Sweden | -$2,247.66 | $788.01 |
| 6 | Germany | -$1,881.33 | $661.21 |
| 7 | Ireland | -$2,843.62 | $994.31 |
| 8 | Singapore | -$1,099.18 | $390.46 |
| 9 | Australia | -$781.85 | $280.62 |
| 10 | Netherlands | -$2,734.45 | $956.52 |
| 11 | Belgium | -$1,276.71 | $451.91 |
| 12 | Finland | -$1,621.03 | $571.10 |
| 13 | United Kingdom | -$863.59 | $308.91 |
| 14 | New Zealand | -$695.38 | $250.69 |
| 15 | United Arab Emirates | -$1,707.11 | $600.90 |
| 16 | Canada | -$1,200.09 | $425.39 |
| 17 | Republic of Korea | -$357.74 | $133.81 |
| 18 | United States of America | -$1,902.47 | $668.52 |
| 19 | Luxembourg | -$2,148.66 | $753.74 |
| 20 | Austria | -$1,477.63 | $521.46 |
| 21 | Slovenia | -$923.18 | $329.54 |
| 22 | Japan | -$857.46 | $306.79 |
| 23 | Israel | -$310.92 | $117.60 |
| 24 | Malta | -$745.63 | $268.08 |
| 25 | Spain | -$1,054.49 | $374.99 |
| 26 | France | -$876.41 | $313.35 |
| 27 | Cyprus | -$740.14 | $266.18 |
| 28 | Italy | -$1,264.65 | $447.74 |
| 29 | Estonia | -$853.47 | $305.41 |
| 30 | Czech Republic | -$1,780.77 | $626.40 |
| 31 | Greece | -$389.28 | $144.73 |
| 32 | Bahrain | -$228.54 | $89.09 |
| 33 | Poland | -$714.44 | $257.28 |
| 34 | Latvia | -$1,660.43 | $584.74 |
| 35 | Lithuania | -$1,681.40 | $592.00 |
| 36 | Croatia | -$1,192.09 | $422.62 |
| 37 | Qatar | -$1,339.97 | $473.81 |
| 38 | Saudi Arabia | -$65.13 | $32.52 |
| 39 | Portugal | -$940.10 | $335.40 |
| 40 | Chile | -$952.03 | $339.53 |
| 41 | Slovakia | -$2,063.35 | $724.21 |
| 42 | Turkey | -$791.17 | $283.84 |
| 43 | Hungary | -$383.78 | $142.82 |
| 44 | Kuwait | -$221.42 | $86.62 |
| 45 | Montenegro | -$1,513.82 | $533.99 |
| 46 | Saint Kitts and Nevis | -$330.65 | $124.43 |
| 47 | Uruguay | -$102.57 | $45.48 |
| 48 | Romania | -$188.76 | $75.32 |
| 49 | Antigua and Barbuda | -$260.41 | $100.12 |
| 50 | Brunei Darussalam | -$4,046.38 | $1,410.65 |
| 51 | Bahamas | -$835.68 | $299.25 |
| 52 | Panama | -$56.82 | $29.65 |
| 53 | Oman | -$96.16 | $43.26 |
| 54 | Georgia | -$34.08 | $21.77 |
| 55 | Trinidad and Tobago | -$85.47 | $39.56 |
| 56 | Malaysia | -$178.70 | $71.83 |
| 57 | Costa Rica | -$87.25 | $40.18 |
| 58 | Serbia | -$113.04 | $49.11 |
| 59 | Thailand | -$90.26 | $41.22 |
| 60 | Kazakhstan | -$133.86 | $56.31 |
| 61 | Belarus | -$149.74 | $61.81 |
| 62 | Bulgaria | -$495.92 | $181.64 |
| 63 | Palau | -$881.58 | $315.14 |
| 64 | Mauritius | -$305.53 | $115.74 |
| 65 | Grenada | -$36.45 | $22.60 |
| 66 | Albania | -$82.19 | $38.43 |
| 67 | China | -$17.85 | $16.16 |
| 68 | Armenia | -$23.58 | $18.14 |
| 69 | Mexico | -$173.74 | $70.12 |
| 70 | Iran (Islamic Republic of) | -$372.29 | $138.84 |
| 71 | Sri Lanka | -$188.22 | $75.13 |
| 72 | Saint Vincent and the Grenadines | -$493.96 | $180.96 |
| 73 | Dominica | -$190.81 | $76.03 |
| 74 | Ecuador | -$41.43 | $24.32 |
| 75 | North Macedonia | -$595.66 | $216.17 |
| 76 | Republic of Moldova | -$58.41 | $30.19 |
| 77 | Peru | -$283.74 | $108.19 |
| 78 | Maldives | -$11.92 | $14.10 |
| 79 | Azerbaijan | -$126.08 | $53.62 |
| 80 | Brazil | -$15.28 | $15.27 |
| 81 | Colombia | -$251.68 | $97.10 |
| 82 | Libya | -$179.72 | $72.19 |
| 83 | Algeria | -$97.79 | $43.83 |
| 84 | Turkmenistan | -$153.81 | $63.22 |
| 85 | Guyana | -$24.62 | $18.50 |
| 86 | Mongolia | -$23.24 | $18.02 |
| 87 | Dominican Republic | -$74.13 | $35.64 |
| 88 | Tonga | -$69.85 | $34.16 |
| 89 | Jordan | -$17.65 | $16.08 |
| 90 | Ukraine | -$66.91 | $33.14 |
| 91 | Tunisia | -$136.17 | $57.11 |
| 92 | Paraguay | -$73.38 | $35.38 |
| 93 | Fiji | -$21.69 | $17.48 |
| 94 | Egypt | -$40.21 | $23.90 |
| 95 | Vietnam | $0.82 | $9.69 |
| 96 | Saint Lucia | -$123.46 | $52.71 |
| 97 | Lebanon | -$3,385.19 | $1,181.77 |
| 98 | South Africa | -$126.89 | $53.90 |
| 99 | Indonesia | -$13.90 | $14.79 |
| 100 | Philippines | -$30.18 | $20.42 |
| 101 | Botswana | -$166.23 | $67.52 |
| 102 | Jamaica | -$28.48 | $19.83 |
| 103 | Samoa | -$330.06 | $124.23 |
| 104 | Kyrgyzstan | -$16.23 | $15.59 |
| 105 | Belize | -$300.80 | $114.10 |
| 106 | Bolivia | -$6.68 | $12.29 |
| 107 | Morocco | -$141.30 | $58.89 |
| 108 | Gabon | -$187.92 | $75.03 |
| 109 | Suriname | -$229.66 | $89.48 |
| 110 | Bhutan | -$16.09 | $15.55 |
| 111 | El Salvador | -$169.40 | $68.61 |
| 112 | Iraq | -$19.98 | $16.89 |
| 113 | Bangladesh | $0.07 | $9.95 |
| 114 | Nicaragua | -$23.24 | $18.02 |
| 115 | Cabo Verde | -$57.15 | $29.76 |
| 116 | Equatorial Guinea | -$128.83 | $54.57 |
| 117 | India | -$1.63 | $10.54 |
| 118 | Micronesia (Federated States of) | -$17.19 | $15.93 |
| 119 | Guatemala | -$33.90 | $21.71 |
| 120 | Honduras | -$27.93 | $19.64 |
| 121 | Lao People's Democratic Republic | -$41.12 | $24.21 |
| 122 | Vanuatu | -$49.14 | $26.99 |
| 123 | Sao Tome and Principe | -$5.09 | $11.74 |
| 124 | Namibia | -$35.76 | $22.36 |
| 125 | Ghana | -$15.04 | $15.18 |
| 126 | Kenya | -$8.09 | $12.78 |
| 127 | Nepal | $2.12 | $9.24 |
| 128 | Cambodia | -$7.34 | $12.52 |
| 129 | Congo | -$162.66 | $66.28 |
| 130 | Angola | -$46.99 | $26.24 |
| 131 | Cameroon | -$54.02 | $28.67 |
| 132 | Zambia | -$1.35 | $10.44 |
| 133 | Papua New Guinea | -$6.41 | $12.19 |
| 134 | Solomon Islands | -$84.69 | $39.29 |
| 135 | Haiti | -$38.08 | $23.16 |
| 136 | Uganda | -$8.47 | $12.91 |
| 137 | Nigeria | -$14.51 | $15.00 |
| 138 | Rwanda | -$572.13 | $208.02 |
| 139 | Togo | -$25.73 | $18.88 |
| 140 | Pakistan | -$18.81 | $16.49 |
| 141 | Mauritania | -$16.12 | $15.55 |
| 142 | Cote d'Ivoire | -$61.59 | $31.30 |
| 143 | United republic of Tanzania | $1.56 | $9.44 |
| 144 | Lesotho | -$36.37 | $22.56 |
| 145 | Senegal | -$109.90 | $48.02 |
| 146 | Sudan | -$212.78 | $83.63 |
| 147 | Djibouti | -$17.70 | $16.10 |
| 148 | Malawi | -$9.43 | $13.24 |
| 149 | Benin | $0.80 | $9.70 |
| 150 | Gambia | -$30.50 | $20.54 |
| 151 | Ethiopia | -$23.09 | $17.97 |
| 152 | Madagascar | $2.66 | $9.06 |
| 153 | Guinea-Bissau | -$27.23 | $19.40 |
| 154 | Guinea | $1.87 | $9.33 |
| 155 | Mozambique | $1.75 | $9.37 |
| 156 | Sierra Leone | -$26.28 | $19.07 |
| 157 | Burkina Faso | -$190.42 | $75.89 |
| 158 | Burundi | -$3.93 | $11.34 |
| 159 | Mali | -$78.53 | $37.16 |
| **Incorporated some imputed data** | | | |
| 160 | Hong Kong, China (SAR) | -$88.74 | $40.69 |
| 161 | Liechtenstein | -$947.29 | $337.88 |
| 162 | Andorra | -$1,351.12 | $477.67 |
| 163 | San Marino | -$1,679.35 | $591.29 |
| 164 | Argentina | -$171.25 | $69.26 |
| 165 | Russian Federation | -$206.59 | $81.49 |
| 166 | Barbados | -$169.31 | $68.59 |
| 167 | Seychelles | -$513.18 | $187.62 |
| 168 | Bosnia and Herzegovina | -$480.42 | $176.27 |
| 169 | Cuba | -$25.51 | $18.81 |
| 170 | Marshall Islands | -$24.20 | $18.35 |
| 171 | Uzbekistan | -$24.65 | $18.51 |
| 172 | Palestine, State of | -$1,100.46 | $390.90 |
| 173 | Venezuela | -$19.32 | $16.67 |
| 174 | Nauru | -$24.47 | $18.45 |
| 175 | Tajikistan | -$9.80 | $13.37 |
| 176 | Tuvalu | -$20.48 | $17.06 |
| 177 | Kiribati | -$24.46 | $18.44 |
| 178 | Swaziland | -$78.08 | $37.01 |
| 179 | Myanmar | -$3.05 | $11.03 |
| 180 | Comoros | -$14.28 | $14.92 |
| 181 | Timor-Leste | -$3.48 | $11.18 |
| 182 | Syrian Arab Republic | -$33.80 | $21.68 |
| 183 | Zimbabwe | -$256.77 | $98.86 |
| 184 | Eritrea | -$2.54 | $10.85 |
| 185 | Liberia | -$0.10 | $10.01 |
| 186 | Democratic Republic of the Congo | -$12.52 | $14.31 |
| 187 | Afghanistan | -$26.99 | $19.32 |
| 188 | Yemen | -$117.02 | $50.48 |
| 189 | Chad | -$22.42 | $17.74 |
| 190 | Niger | -$13.39 | $14.61 |
| 191 | Central African Republic | -$12.93 | $14.45 |
| 192 | South Sudan | -$41.83 | $24.46 |
| 193 | Somalia | -$124.10 | $52.93 |

At the lower bound of the effect size (0.74), coated suture was associated with increased costs in Bangladesh,

Benin, Guinea, Madagascar, Mozambique, United republic of Tanzania, and Vietnam.

In 186 it could reduce the costs or increase the costs. At the upper bound (1.09), coated suture was associated with an increase in costs in all countries.

# **Appendix S3: Sensitivity analysis results**

Table S5: Difference in costs associated with an SSI when the cost of coated suture was increased by 50%.

| **HDI group** | **Wound category** | **Coated suture group** | **Uncoated suture group** | **Cost difference per patient** |
| --- | --- | --- | --- | --- |
| High HDI | All wounds | $1,400 to $2,037 | $1,848 | -$448 to $189 |
|  | Clean-contaminated wounds | $794 to $1,144 | $1,028 | -$235 to $115 |
|  | Contaminated-dirty wounds | $2,050 to $2,994 | $2,726 | -$676 to $268 |
| Middle HDI | All wounds | $139 to $180 | $144 | -$5 to $36 |
|  | Clean-contaminated wounds | $96 to $117 | $86 | $10 to $31 |
|  | Contaminated-dirty wounds | $196 to $263 | $221 | -$25 to $43 |
| Low HDI | All wounds | $170 to $226 | $186 | -$16 to $40 |
|  | Clean-contaminated wounds | $99 to $121 | $90 | $9 to $31 |
|  | Contaminated-dirty wounds | $274 to $379 | $326 | -$52 to $52 |

HDI stands for Human Development Index. *Negative is cost saving due to use of coated suture, positive is cost increase.

Table S6: Difference in costs associated with an SSI when the cost of coated suture was decreased by 50%.

| **HDI group** | **Wound category** | **Coated suture group** | **Uncoated suture group** | **Cost difference per patient** |
| --- | --- | --- | --- | --- |
| High HDI | All wounds | $1,365 to $2,002 | $1,848 | -$483 to $155 |
|  | Clean-contaminated wounds | $759 to $1,109 | $1,028 | -$270 to $80 |
|  | Contaminated-dirty wounds | $2,015 to $2,959 | $2,726 | -$711 to $233 |
| Middle HDI | All wounds | $104 to $145 | $144 | -$40 to $1 |
|  | Clean-contaminated wounds | $61 to $82 | $86 | $25 to -$4 |
|  | Contaminated-dirty wounds | $161 to $228 | $221 | -$60 to $8 |
| Low HDI | All wounds | $135 to $191 | $186 | -$51 to $5 |
|  | Clean-contaminated wounds | $64 to $86 | $90 | -$26 to -$4 |
|  | Contaminated-dirty wounds | $239 to $344 | $326 | -$87 to $17 |

HDI stands for Human Development Index. *Negative is cost saving due to use of coated suture, positive is cost increase.

Table S7: Difference in costs associated with an SSI when the cost of uncoated suture was increased by 50%.

| **HDI group** | **Wound category** | **Coated suture group** | **Uncoated suture group** | **Cost difference per patient** |
| --- | --- | --- | --- | --- |
| High HDI | All wounds | $1,382 to $2,019 | $1,862 | -$480 to $157 |
|  | Clean-contaminated wounds | $776 to $1,126 | $1,043 | -$267 to $83 |
|  | Contaminated-dirty wounds | $2,032 to $2,976 | $2,740 | -$708 to $236 |
| Middle HDI | All wounds | $121 to $162 | $158 | -$37 to $3 |
|  | Clean-contaminated wounds | $78 to $99 | $101 | -$22 to -$2 |
|  | Contaminated-dirty wounds | $178 to $245 | $235 | -$57 to $10 |
| Low HDI | All wounds | $152 to $208 | $201 | -$48 to $7 |
|  | Clean-contaminated wounds | $81 to $103 | $104 | -$23 to -$1 |
|  | Contaminated-dirty wounds | $256 to $361 | $341 | -$85 to $20 |

HDI stands for Human Development Index. *Negative is cost saving due to use of coated suture, positive is cost increase.

Table S8: Difference in costs associated with an SSI when the cost of uncoated suture was decreased by 50%.

| **HDI group** | **Wound category** | **Coated suture group** | **Uncoated suture group** | **Cost difference per patient** |
| --- | --- | --- | --- | --- |
| High HDI | All wounds | $1,382 to $2,019 | $1,834 | -$452 to $185 |
|  | Clean-contaminated wounds | $776 to $1,126 | $1,015 | -$239 to $111 |
|  | Contaminated-dirty wounds | $2,032 to $2,976 | $2,712 | -$680 to $264 |
| Middle HDI | All wounds | $121 to $162 | $130 | -$9 to $31 |
|  | Clean-contaminated wounds | $78 to $99 | $73 | $6 to $26 |
|  | Contaminated-dirty wounds | $178 to $245 | $207 | -$29 to $38 |
| Low HDI | All wounds | $152 to $208 | $173 | -$20 to $35 |
|  | Clean-contaminated wounds | $81 to $103 | $76 | $5 to $27 |
|  | Contaminated-dirty wounds | $256 to $361 | $313 | -$57 to $48 |

HDI stands for Human Development Index. *Negative is cost saving due to use of coated suture, positive is cost increase.

Table S9: Difference in costs associated with SSI when the proportion of hospital stay cost to all healthcare costs was adjusted to 0.88.

| **Patient category** | **SSI cost per patient with a coated suture** | **SSI cost per patient with an uncoated suture** | **SSI related cost difference per patient*** |
| --- | --- | --- | --- |
| High HDI countries: all wounds | $1 480 to $2 163 | $1,980 | -$500 to $183 |
| High HDI countries: clean-contaminated wounds | $825 to $1 199 | $1,096 | -$270 to $104 |
| High HDI countries: contaminate-dirty wounds | 2 187 to $3 204 | $2,935 | -$749 to $269 |
| Middle HDI countries: all wounds | $127 to $171 | $152 | -$25 to $19 |
| Middle HDI countries: clean-contaminated wounds | $81 to $90 | $90 | -$9 to $13 |
| Middle HDI countries: contaminate-dirty wounds | $188 to $234 | $234 | -$46 to $26 |
| Low HDI countries: all wounds | $163 to $223 | $200 | -$37 to $23 |
| Low HDI countries: clean-contaminated wounds | $85 to $109 | $95 | -$10 to $13 |
| Low HDI countries: contaminate-dirty wounds | $276 to $390 | $353 | -$77 to $37 |

HDI stands for Human Development Index. *Negative is cost saving due to use of coated suture, positive is cost increase.

Table S10: Difference in costs associated with SSI when the proportion of hospital stay cost to all healthcare costs was adjusted to 0.96.

| **Patient category** | **SSI cost per patient with a coated suture** | **SSI cost per patient with an uncoated suture** | **SSI related cost difference per patient*** |
| --- | --- | --- | --- |
| High HDI countries: all wounds | $1 279 to $1 867 | $1,709 | -$430 to $159 |
| High HDI countries: clean-contaminated wounds | $723 to $1 049 | $958 | -$234 to $91 |
| High HDI countries: contaminate-dirty wounds | $1 868 to $2 736 | $2,505 | -$637 to $230 |
| Middle HDI countries: all wounds | $113 to $150 | $133 | -$20 to $17 |
| Middle HDI countries: clean-contaminated wounds | $75 to $93 | $81 | -$6 to $12 |
| Middle HDI countries: contaminate-dirty wounds | $165 to $226 | $203 | -$38 to $23 |
| Low HDI countries: all wounds | $141 to $192 | $171 | -$30 to $20 |
| Low HDI countries: clean-contaminated wounds | $76 to $96 | $84 | -$7 to $12 |
| Low HDI countries: contaminate-dirty wounds | $236 to $331 | $299 | -$63 to $32 |

HDI stands for Human Development Index. *Negative is cost saving due to use of coated suture, positive is cost increase.

Table S11: Best case scenario

| **HDI group** | **Patient category** | **Coated suture group** | **Uncoated suture group** | **Cost difference per patient** |
| --- | --- | --- | --- | --- |
| High HDI | All wounds | $1,467 | $2,001 | -$533 |
|  | Clean-contaminated wounds | $811 | $1,113 | -$303 |
|  | Contaminated-dirty wounds | $2,177 | $2,960 | -$783 |
| Middle HDI | All wounds | $111 | $168 | -$57 |
|  | Clean-contaminated wounds | $65 | $106 | -$41 |
|  | Contaminated-dirty wounds | $173 | $251 | -$78 |
| Low HDI | All wounds | $146 | $215 | -$69 |
|  | Clean-contaminated wounds | $68 | $110 | -$42 |
|  | Contaminated-dirty wounds | $260 | $369 | -$109 |

HDI stands for Human Development Index. *Negative is cost saving due to use of coated suture, positive is cost increase.

Table S12: Worst case scenario

| **HDI group** | **Patient category** | **Coated suture group** | **Uncoated suture group** | **Cost difference per patient** |
| --- | --- | --- | --- | --- |
| High HDI | All wounds | $1,905 | $1,713 | $192 |
|  | Clean-contaminated wounds | $1,077 | $953 | $124 |
|  | Contaminated-dirty wounds | $2,785 | $2,520 | $265 |
| Middle HDI | All wounds | $170 | $122 | $49 |
|  | Clean-contaminated wounds | $112 | $69 | $44 |
|  | Contaminated-dirty wounds | $248 | $193 | $55 |
| Low HDI | All wounds | $212 | $160 | $52 |
|  | Clean-contaminated wounds | $115 | $71 | $44 |
|  | Contaminated-dirty wounds | $352 | $289 | $64 |

HDI stands for Human Development Index. *Negative is cost saving due to use of coated suture, positive is cost increase.

Figure S1: Cost savings/increases by HDI rank for all wounds. Blue (1.09 effect size) and green (0.74 effect size)

Figure S2: Top 10 countries with cost savings (lower bound, 0.74). A negative cost indicates cost savings

Figure S3: Bottom 10 countries with cost savings/increase in costs (lower bound, 0.74). A negative cost indicates cost savings.

Figure S4: Top 10 countries with increase in costs (upper bound, 1.09)

Figure S5: Bottom 10 countries with increase in costs (upper bound, 1.09)

# **Appendix S4: Discussion**

This analysis relied much on data from the multi-centre, cohort study that measured the incidence of SSI among 12 patients undergoing elective or emergency gastrointestinal resection in high, middle, and low-HDI countries (GlobalSurg 2). The major limitation of GlobalSurg 2 was lost to follow-up of the patients at the 30-day follow-up period especially in Global South. However, sensitivity analysis comparing the results at the time of discharge and the 30-day follow-up period showed that the results were not affected by the loss to follow-up. Further, in the study there was possibility of missing some patients during the follow-up which of course was minimized by using local collaborators.
